# Ambiguous handedness and visuospatial pseudoneglect in schizotypy in physical and computer-generated virtual environments

**DOI:** 10.1038/s41598-022-16454-2

**Published:** 2022-07-16

**Authors:** János Kállai, Tamás Páll, Róbert Herold, Tamás Tényi, András Norbert Zsidó

**Affiliations:** 1 Institute of Behavioral Sciences, Medical Faculty University of Pécs, Pécs, Hungary; 2 Artistic Research at the University of Applied Arts Vienna, Vienna, Austria; 3 Department of Psychiatry and Psychotherapy, Medical School, University of Pécs, Pécs, Hungary; 4 Institute of Psychology, Arts and Sciences Faculty, University of Pécs, Pécs, Hungary; 5 Institute of Behavioral Sciences, Medical School, University of Pécs, 7624 Szigeti Street 12, Pécs, Hungary

**Keywords:** Neuroscience, Psychology

## Abstract

Virtual reality (VR) technology has increased clinical attention in the health care of schizophrenia spectrum disorders in both diagnoses of the symptoms and assessment of schizotypal traits. However, the exact nature of VR-induced positive treatment effect in schizotypy is still unknown. In this study, VR technology was used as a non-invasive neurocognitive trigger to test the asymmetric visuospatial representational instability found in individuals with high schizotypy. The study aimed to reveal the brain functional hemispheric laterality in physical and virtual realities in individuals with schizotypal traits. Fifty-one healthy, right-handed participants (24 males and 27 females) were enrolled through public advertisements. Hemispheric functional asymmetry was measured by the Line Bisection Task (LBT). The results revealed that (a) LBT bias in the physical reality showed a handedness-related leftward pseudoneglect, however, similar handedness-related pseudoneglect in VR has not been found. (b) Comparing LBT bias in physically real and VR environments showed rightward drift in VR environments independently to the degree of handedness. (c) The schizotypy has no association with handedness, however, the cognitive schizotypy is related to the LBT bias. Higher cognitive schizotypy in VR associated with left hemispatial pseudoneglect. In conclusion, schizotypy is associated with ambiguous behavioral and cognitive functional laterality. In individuals with high cognitive schizotypy, the VR environment enhanced the representational articulation of the left hemispace. This effect may be originated from the enhancement of the right hemisphere overactivation and is followed by a lower mental control of the overt behavior.

## Introduction

Schizotypy is described as a neurodevelopmental and psychological vulnerability for schizophrenia across a wide continuum ranging from normal adaptation to full-blown schizophrenia. Schizotypy is a bio-socially determined^1–3^ multidimensional syndrome that manifests in cognitive (positive), interpersonal (negative, affective), and disorganization (behavioural) symptoms that in part rely on ambiguous brain hemispheric functions. In schizotypy, the common asymmetric hemispheric functional patterns that are detected in healthy right-handed individuals seem to be imbalanced^4^. In schizophrenia spectrum disorders (involving schizotypy), reduced left hemisphere dominance can be found for language, and reduced right hemisphere dominance can be detected for face processing^5–10^. This atypical lateralisation induces an unusual cognitive processing style that enhances the odd or, in other cases, a creative view of the physical and social environment^11–14^; moreover, it is associated with lateral hemispherical dopaminergic dysfunction that in exaggerated cases, may be considered as a disposition for manifestation of the main schizotypal dysfunction. It is also linked to neurocognitive specialties^15^ such as the inability to express pleasure, impaired body awareness, aberrant salience, and psychotic-like symptoms^16–18^ that have been found to carry predictive effect on the development of schizophrenia spectrum disorders^19^. Ambiguous handedness is frequently detected with different Cohen’s d effect sizes in schizophrenia spectrum disorders and persons with non-clinical schizotypal tendencies and siblings of schizophrenia patients^20–23^. Ambiguous handedness in schizotypy is significantly higher than in non-schizotypy population^24^. However, the direction and rate of manifestation, and the aetiology of imbalanced hemispheric laterality in schizotypy are currently under debate. The line bisection task (LBT) and its completely identical computer-generated virtual reality (VR) format were used in this study. A large number of studies on schizotypy and pseudoneglect have been reported with different results. In a healthy population, a decreased leftward LBT bias correlated with high schizotypy involving the magical thinking subscale^25^, and a similar decreased leftward LBT bias was found in right-handed schizophrenic patients^26,27^. However, in other studies with schizotypy involving the magical thinking scale a strong leftward LBT bias was found^28,29^.

### Line bisection and pseudoneglect

In healthy right-handed individuals, the visuospatial laterality tests show a systematic left hemispace advantage while the right hemispace is partly neglected. The visuospatial perceptual load induces a functional elevation in the right frontoparietal attention network, contrasting to the left one. Consequently, the contralateral hemispace is perceptually sharpened and overrepresented^30,31^. The most extensively used paper and pencil method to test the hemispheric asymmetry of visuospatial attention is the LBT. The magnitude and the direction of the bisection error bias in neurologically intact individuals for most cases induce a leftward bias in contrast to clinical cases with right parietal lobe lesions which induces an opposite LBT bias that is ipsilateral to the lesioned hemisphere. In a healthy person, the LBT error bias is defined as the representational deviation in the tested hemispace which is related to a brain asymmetric functional characteristic without lesions in the brain tissues. In neurological cases, the bisection error indicates a functional consequence of an anatomical impairment in a specific area of the brain^32^. A large body of evidence has confirmed that LBT involves two different attentional mechanisms: person-centred lateral attention allocation and object-centred selective attention^33,34^. Several studies have demonstrated that healthy individuals show consistent leftward pseudoneglect on LBT^35^. The rate of pseudoneglect depends on handedness, sex, age, size of the bisected objects, and the method of bisection, and is specific to the attention allocation in right or left hemispace^36–39^. There is evidence for the existence of a leftward bias in LBT in healthy participants; however, the left and right representations of hemispace provide an integrated or competitive visuospatial environment. Competition for attentional resources interferes with multimodal integration by causing mismatches or dissociation in higher-order representations in patients and healthy individuals. Neuropsychological and functional magnetic resonance imaging (fMRI) studies with LBT have shown state or trait-dependent individual neural network variations in pseudoneglect. Nonetheless, LBT can be considered a validated measurement for assessing lateral cerebral basis of spatial attention allocation. However, the task types and the participants’ visual scanning habits, sex, and handedness should also be considered when evaluating experimental data^40–42^.

### Hypothesis

The application of VR technology adds ambiguity to the construction of real background for intended action as the immersing participant must select between two visuospatial frameworks where the goal-directed action can be realised. VR generates two realities simultaneously: physical reality (PR) and virtual reality (VR). Consonant neurocognitive research data^22,32^ have supported that motor (handedness) and visuospatial construction of reality in schizotypy is likely to the right hemisphere activity (in right-handed individuals) but the functional specificity of hemispheres for the reality generation in schizotypy is ambiguous. Consequently, during the selection between the two visuospatial frameworks, the right hemisphere in schizotypy is available for reality construction but only at an ambiguous rate. A previous study revealed that induction of VR in healthy participants modifies the hemispheric activity-dependent rate of the LBT bias in the left hemispace of the environment^43^. Nowadays the role of VR is using in work, social media, and gaming in the digital environment is gradually increasing. A large body of evidence indicates that individuals with schizotypal traits have an elevated vulnerability to reality distortions that are frequently associated with problematic internet usage and computer gaming dependencies^3,14,19^. However, the effect of the VR on the brain's hemispheric functions where the mental construction of reality is taking place in schizotypy is debated. The present study aimed to reveal the brain functional hemispheric laterality in physical and computer-generated VR environments in individuals with schizotypal traits. We hypothesized that VR-triggered visuospatial activation in schizotypy with imbalanced function laterality induces compensative cognitive enhancement in the right hemisphere, which would be expressed by the LBT error bias difference comparing PR and VR hemispace representational biases.

## Methods

### Participants

Because left- and right-handed individuals exhibit distinct patterns on visuospatial tasks and visual scan measurements^44^, only right-handed individuals were recruited for this investigation. Fifty-one healthy, right-handed college students and recent graduates [24 males (mean age = 26, SD = 2.1) and 27 females (mean age = 22.5, SD = 2.7)] were enrolled as participants through public advertisements. A priori sample size analysis using the G*Power3 software^45^ for the most complex GLM (Model 3) we used during data analyses (with f = 0.4, 1-beta = 0.8) showed that the required sample size was 51. Handedness was measured by the Edinburgh Handedness Inventory (with 10 items) (EHI)^46^, which is a frequently used method in clinical practice. The Handedness Laterality Index (EHI) was calculated {LQ = [(Right-Left)/[(Right + Left)] × 100}. The inclusion criterion for LQ was a score of 70 or higher. Based on the EHI^47^ the degree of handedness was located on a consistent–inconsistent right-handedness continuum as a metrical variable (from 100 to 70%). The handedness mean was 93, with a standard deviation of 7.8 (range 70–100). There were no significant sex differences in handedness [males N = 24, mean (SD) = 93.0 (5.5); females N = 27, mean (SD) = 96.6 (4.8); t = 1.76, ns]. No participants had any previous psychiatric or neurologic illness or any prior experience with VR. Participants received a small incentive for taking part in the study. Informed consent was obtained from all subjects and the investigation was conducted in adherence to the principles of the Declaration of Helsinki and approved by the Regional Research Ethics Committee of the Medical Centre of the University of Pécs.

### Apparatus and procedures

The computer-generated virtual environment was created in the Unity game engine, version 2019.3f1. The target hardware for rendering was a wireless HTC Vive 2018 head-mounted display (HMD) and its controller. The user interface was created with Unity’s own UI kit, which adjusts the interface to proportionally conform to all screen sizes. The kit provided a custom user interface designed specifically for laboratory experiments. The software used a hybrid virtual-physical calibration approach to fit the coordinate system of the virtual space in its physical location. On the first screen (the calibration and data input screen), experimenters could calibrate the virtual room with the HTC Vive controllers by entering calibration mode and physically attaching one of the handheld controllers to a cross-shaped target point in the physical room. The physical target point had a virtual double that served as the origin of the virtual space, with the same x–y coordinates. The two points were thus synchronised with millimetre-level precision, fixing the two spaces in one coordinate system. The experimental data, location and length of the lines, and room size were stored locally in JSON files, making them accessible to the laboratory for creation of new virtual setups and circumstances. The data collected from these devices were stored in a CSV file. Data sampling frequency rate of the device was 4 Hz. This programme has been applied in several experiments in our laboratory^46^.

### Procedure

**Figure 1:**
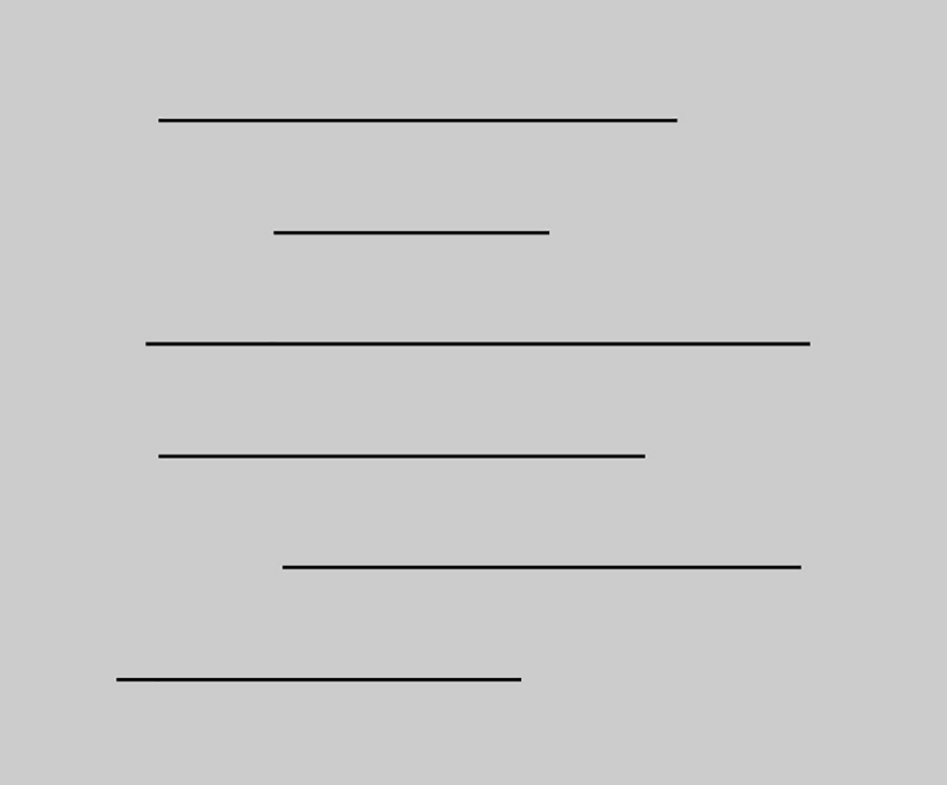
The design of the Line Bisection Task.

Participants were required to bisect six black horizontal lines, which varied from 11 to 27 cm in length, and were placed in different positions on a response sheet (Fig. 1). A similar line setting was reported in previous studies^32,48–52^. The line bisection error (LBE) was calculated as the rate of deviation from the true midpoint of the line and transformed into a percentage (LBE = deviation rate from the middle of the line/true half of the Line × 100). LBT was projected in focal extrapersonal space in both PR and VR environments. Before VR immersion, in a separate room LBT baseline was assessed in a PR setting. The participants stood in front of a screen located 1 m away. LBT consisted of two identical blocks with a 5-s break in between, and the design of the LBT was the same in PR and VR conditions. The number of presented lines and blocks were identical. In the real environment, the Cronbach’s α for 12 bisected lines was 0.860. In the virtual reality environment, the Cronbach’s α for 12 bisected lines was 0.656.

Participants performed the bisection in PR condition using a paper screen and a wooden stick. In the VR condition, the stick was replaced by a laser pointer on the VR controller, which was used to bisect the lines and automatically record the result of bisection. The screen where the lines were presented in PR and VR conditions covered 90 degrees of the participant’s visual field. The rate of bisection errors in both PR and VR conditions was calculated as a percentage. Leftward errors were indexed as negative deviation scores and rightward errors were indexed as positive deviation scores. The same bisection task was implemented in two conditions: LBT applied in a PR environment (PR_LBT, and LBT applied in a VR environment (VR_LBT). Participants’ summary scores in the PR_LBT and VR_LBT conditions were the average directional bisection errors bias.

### Measurement

Schizotypal Personality Questionnaire Brief Revised (SPQ-BR; 52, 48) self-reported measurement was used to reveal vulnerability to schizotypy that is associated with atypical right-hemispheric laterality. Participants used a 5-point Likert-style scoring format that ranged from 0 (strongly disagree) to 4 (strongly agree). The SPQ-BR consists of 32 items that include the cognitive (positives symptoms: ideas of reference/suspiciousness, magical thinking, unusual perceptions), interpersonal (negatives symptoms: no close friends/constricted affects, excessive social anxiety), and behavioural components (disorganisation symptoms: eccentric behaviour, and odd speech) in social adaptation. In the present study, all factors had excellent internal reliability scores (Cronbach α = 0.71–0.90). The SPQ-BR items conform to those observed in schizophrenia spectrum disorders^53,54^ and high scores on SPQ-BR are considered an index for maladaptation in a healthy sample.

### Data analysis

First, we started with two one-sample Student t-tests to examine whether there was a significant pseudoneglect or, rather a rightward drift in the VR condition. The dependent variables were PR_LBT and VR_LBT, the test value was 0. Then, we performed GLMs, setting PR_LBT and VR_LBT error rates as the repeated measures factor. In the first model, we used sex as a between-subjects factor. In the second model, we also added handedness as a covariate. In the third model, besides sex and handedness, we also added the three SPQ scales (cognitive, interpersonal, and disorganised) as covariates. After this, we performed similar GLM analyses on three SPQ subscales where 'setting’ (PR and VR LBT error rates) was the repeated measures factor. Gender, handedness, and three subscales of SPQ cognitive, two subscales of SPQ interpersonal, and two subscales of SPQ disorganised subscale were entered as covariates.

To understand significant covariate main effects and interactions, we conducted additional linear regressions with the same variables in the model. The assumption of normality for the dependent variables was met, Skewness and Kurtosis values were between − 2 and 2. For ANOVA, Levene’s homogeneity tests were nonsignificant. For linear regressions, the Durbin-Watson tests for autocorrelation were nonsignificant, and VIF values were lower than 2. The data for descriptive variables are shown in Table 1.

**Table 1:** Descriptive statistics of the measured variables (N = 51).

	Mean	SD	Min	Max	Skewness	Kurtosis
LQ handedness	94.9	6.99	70.0	100	− 1.71	2.79
LBT error—PR	− 1.55	2.81	− 7.95	3.73	− 0.389	− 0.402
LBT error—VR	0.439	2.99	− 5.10	7.16	0.508	− 0.467
SPQ-BR cognitive	13.9	6.22	2	32	0.586	0.223
SPQ-BR interpersonal	14.3	7.78	1.00	36.0	0.650	0.316
SPQ-BR disorganised	14.9	6.57	4	30	0.238	− 0.944
**Subscales**						
Magical thinking	3.29	3.5	0	13	1.172	0.560
Suspiciousness	8.39	3.4	1	18	0.508	0.753
Unusual perceptions	2.72	3.1	0	13	1.392	2.14
Social anxiety	6.80	3.9	0	16	0.311	− 0.518
Constricted affects	6.82	4.6	0	20	0.810	0.428
Odd speech	8.37	2.7	0	14	− 0.477	0.656
Eccentric behaviour	6.54	4.0	1	16	0.334	− 0.883

Abbreviations: Handedness Laterality Quotient (LQ). Line Bisection Task error bias (negative scores indicate a leftward bias and the positive scores indicate a rightward bias from the middle point of the test line), physical reality (PR), virtual reality (VR), Schizotypy Personality Questionnaire Brief Revised (SPQ-BR), and the Cognitive (involving magical thinking, ideas of reference/suspiciousness, unusual perceptions subscales), Interpersonal (involving excessive social anxiety, no close friends/constricted affects subscales), Disorganised (involving odd speech and eccentric behaviour subscales).

### Ethical approval

Ethical allowance #2017-2022 No.6732 (Regional Committee of University of Pécs).

## Results

The leftward pseudoneglect was evident in the PR condition (t(50) = 3.94, *p* < 0.001, Cohen’s d = 0.551), but we did not find a significant rightward pseudoneglect in the VR condition (t(50) = 1.05, *p* = 0.300, Cohen’s d = 0.147). This was only a rightward drift relative to the PR condition.

The descriptive statistics of the LBT and the schizotypy main factors and subscales are shown in Table 1.

**Figure 2:**
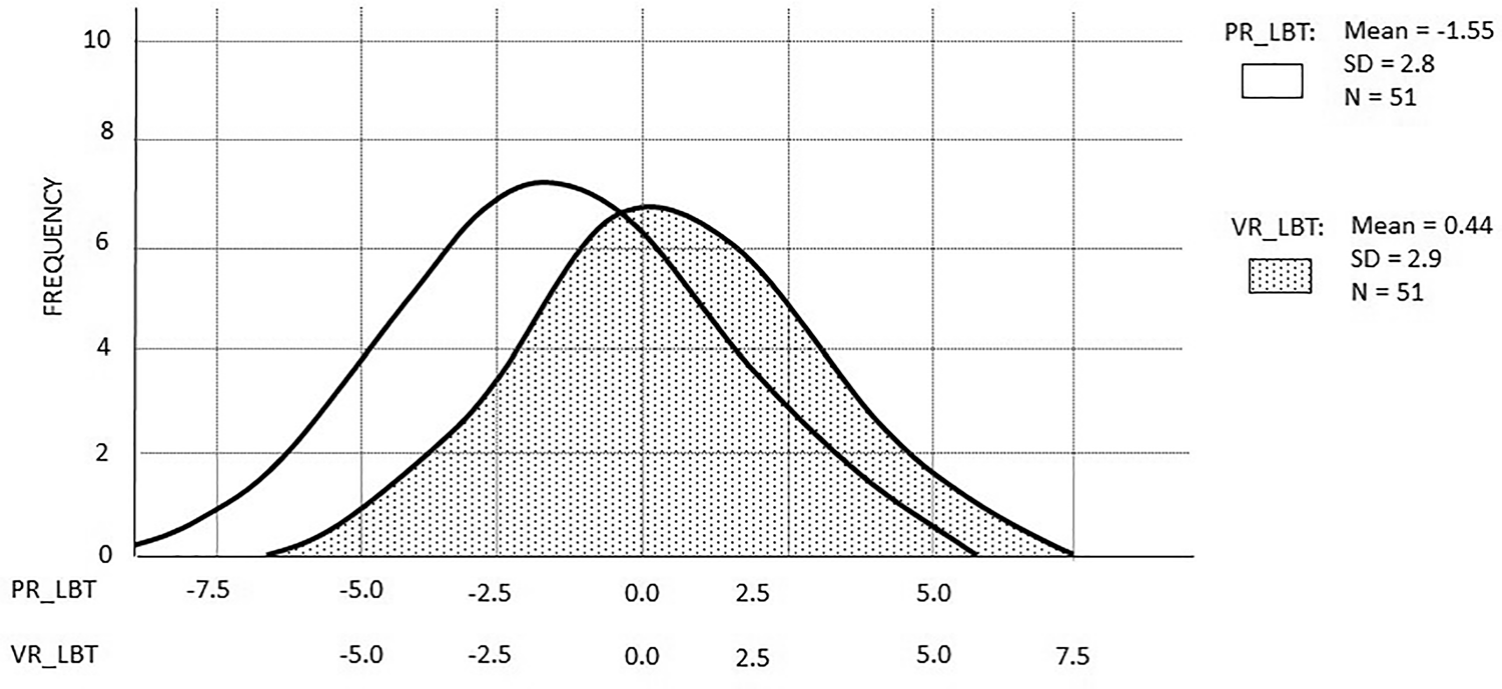
The scattering of the LBT error in a PR and VR environments. Negative scores represent the mean of the leftward bias to the centre (0.0.) of the presented test lines (in percent). In PR, LBT error bias showed a significant leftward pseudoneglect compared to right hemispace. The LBT error bias in VR drifted toward the right side of the hemispace.

In the Model 1, the main effect of ANOVA showed that there is a significant difference between the LBT error rates in PR and VR settings [F(1,49) = 21.2, *p* < 0.001, ɳp2 = 0.3]. Participants showed a leftward bias in the PR setting (M = − 1.55, 95%CI = − 2.34 to − 0.76) and a slightly rightward bias in the VR setting (M = 0.44, 95%CI = − 0.40 to 1.28). Figure 2 shows the distribution of variables. The main effect of gender [F(1,49) = 1.45, *p* = 0.23, ɳp2 = 0.03] and the interaction between error rates and gender [F(1,49) = 0.4, *p* = 0.52, ɳp2 = 0.01] were nonsignificant.

In the Model 2, upon adding handedness as a covariate, only its main effect was significant [F(1,48) = 5.73, *p* = 0.021, ɳp2 = 0.11]. Follow-up linear regression showed a negative relationship between the variables [β = − 0.297, 95%CI = − 0.58 to − 0.016, t(48) = − 2.13, *p* = 0.039], that is, lower handedness scores were associated with a rightward bias, while higher scores were associated with a leftward bias. The main effect of LBT bias between PR and VR settings disappeared [F(1,48) = 0.07, *p* = 0.79, ɳp2 < 0.01] and the gender main effect remained nonsignificant [F(1,48) = 0.39, *p* = 0.54, ɳp2 = 0.01]. The two-way interactions between setting and gender [F(1,48) = 0.37, *p* = 0.55, ɳp2 = 0.01] and setting and handedness [F(1,48) = 0.01, *p* = 0.95, ɳp2 < 0.01] were also nonsignificant.

**Figure 3:**
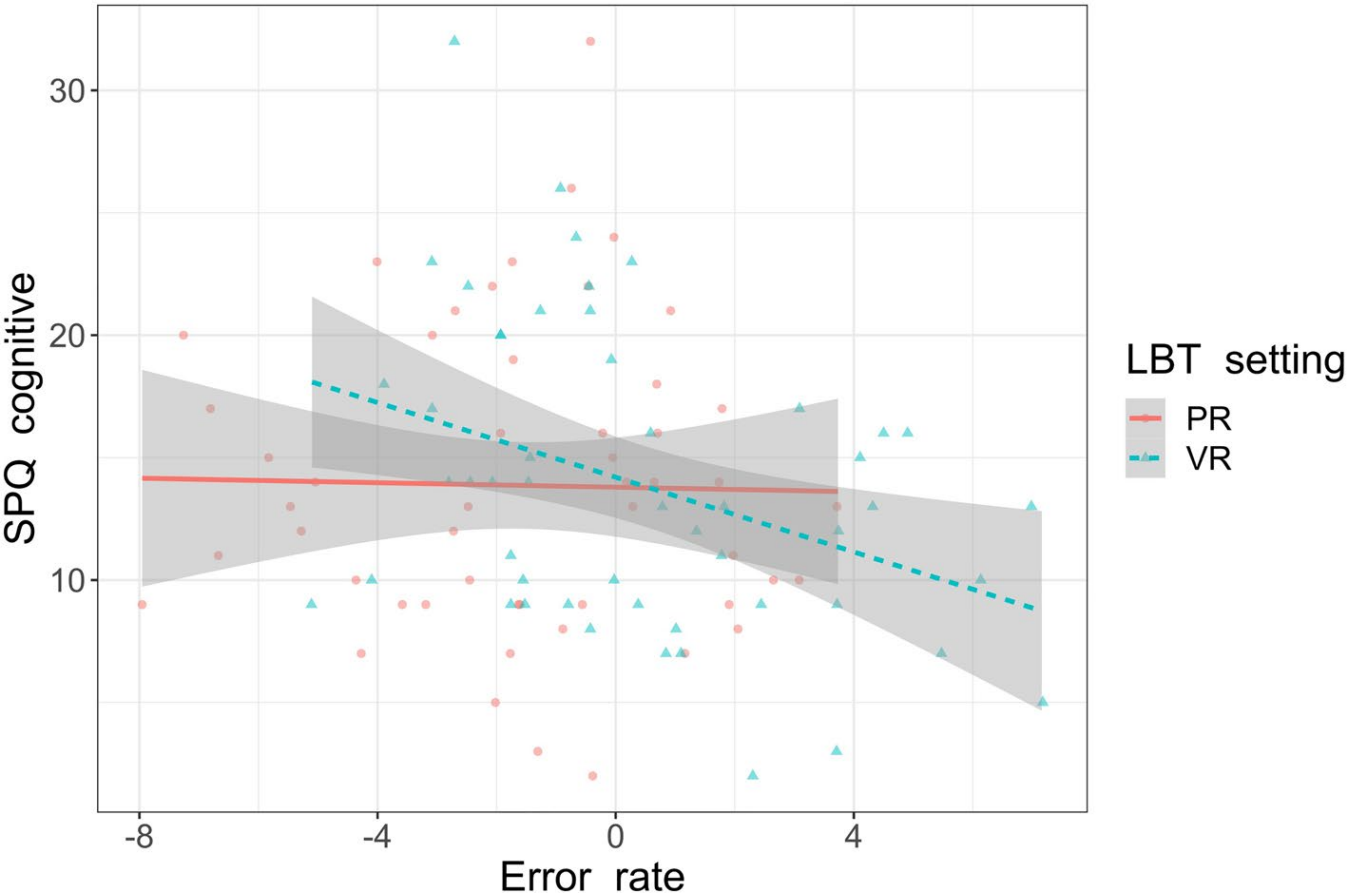
The association between LBT error rates in PR (continuous line) and VR (dashed line) settings and SPQ cognitive. The “0” indicates the centre of the test line. Negative scores indicate leftward LBT error bias and positive cores indicate rightward LBT error bias.

The Model 3 focused on the different scales of schizotypy. The main effects of SPQ cognitive [F(1,45) = 4.57, *p* = 0.038, ɳp2 = 0.09] and handedness were significant [F(1,45) = 7.61, *p* = 0.008, ɳp2 = 0.15]. The main effect of LBT bias between PR and VR settings [F(1,45) = 0.78, *p* = 0.38, ɳp2 = 0.02] and gender remained nonsignificant [F(1,45) = 0.55, *p* = 0.46, ɳp2 = 0.01]. The effects of the SPQ interpersonal [F(1,45) = 1.32, *p* = 0.26, ɳp2 = 0.03] and disorganised [F(1,45) = 0.16, *p* = 0.69, ɳp2 < 0.01] factors were also nonsignificant. Regarding the two-way interactions, the SPQ cognitive factor and LBT settings were significant [F(1,45) = 5.57, *p* = 0.023, ɳp2 = 0.11]. Follow-up linear regressions showed that the cognitive factor was a significant negative predictor of LBT errors in VR [β = − 0.40, 95%CI = − 0.65 to − 0.15, t(45) = − 3.18, *p* = 0.003] but not in PR setting [β = − 0.07, 95%CI = − 0.36 to 0.21, t(44) = − 0.51, *p* = 0.61]. That is, when participants completed the LBT in VR, higher scores on the SPQ cognitive factor resulted in a more pronounced leftward bias on the task. Figure 3 shows this interaction. The other interactions were nonsignificant: setting x SPQ interpersonal [F(1,45) = 3.89, *p* = 0.06, ɳp2 = 0.08], setting x SPQ disorganised [F(1,45) = 0.20, *p* = 0.66, ɳp2 < 0.01], setting x gender [F(1,45) = 0.47, *p* = 0.50, ɳp2 = 0.01], setting x handedness [F(1,45) = 0.08, *p* = 0.89, ɳp2 < 0.01]. Table 1 shows the descriptive statistics for all the variables in the model.

Handedness (F(1,41) = 6.51, *p* = 0.015, ɳp2 = 0.14) and the suspicious subscale of SPQ cognitive (F(1,41) = 6.48, *p* = 0.015, ɳp2 = 0.14) main effects were significant. Follow-up linear regressions showed a negative association between handedness and both real (β = − 0.32, 95%CI = − 0.66 to 0.02) and VR (β = − 0.35, 95%CI = − 0.65 to − 0.05) error rates. We found a similar association between the suspicion subscale and real (β = − 0.38, 95%CI = − 0.74 to − 0.03) and VR (β = − 0.33, 95%CI = − 0.64 to − 0.01) conditions. The other main effects were nonsignificant.

**Figure 4:**
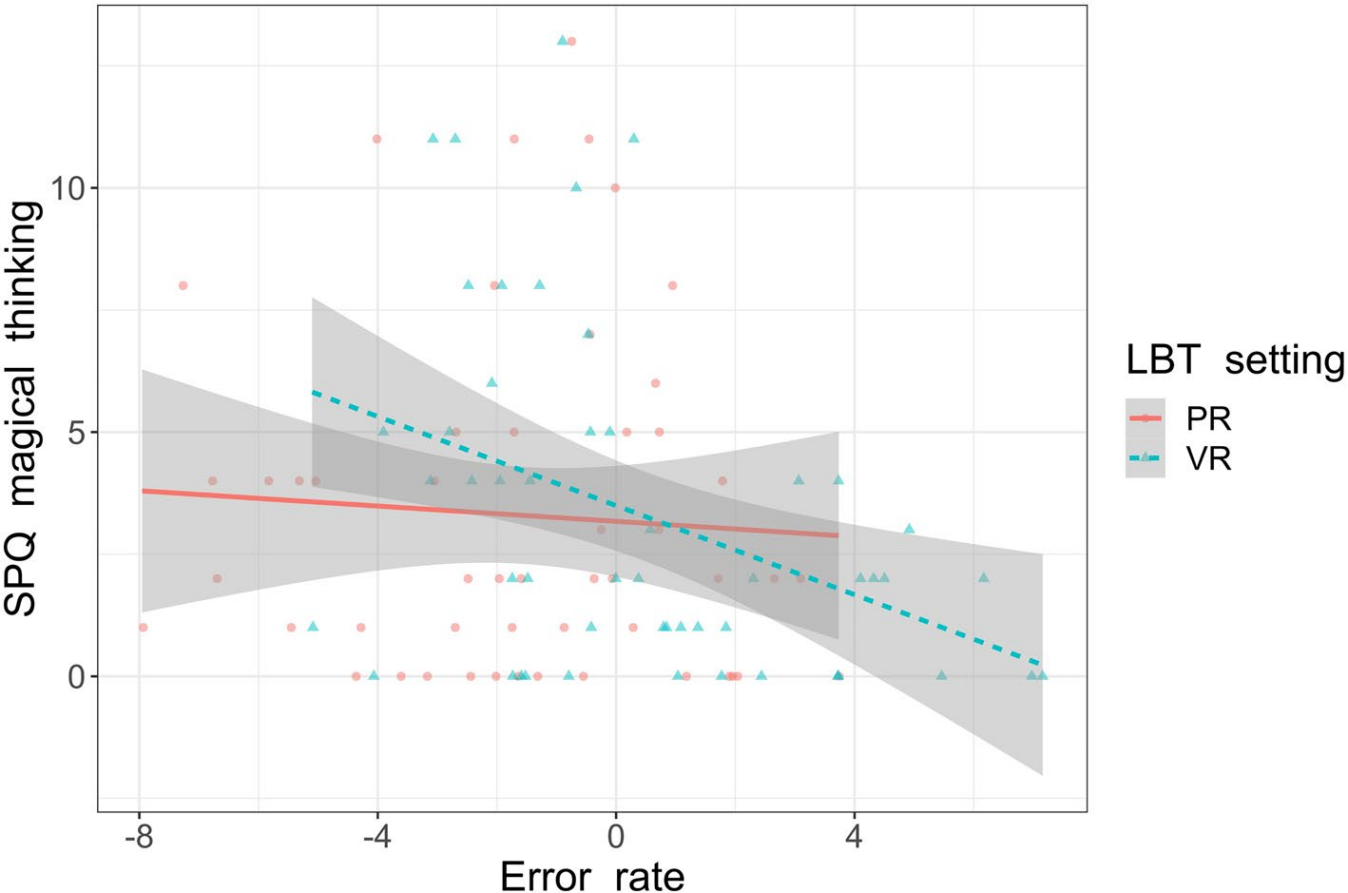
The association between LBT error rates in PR (continuous line) and VR (dashed line) settings and the magical thinking subscale of SPQ-BR. The “0” indicates centre of the line. The negative scores indicate leftward LBT error bias and positive scores indicate rightward LBT error bias.

We also found a significant interaction between the magic subscale of the SPQ cognitive scale and LBT error rates [F(1,41) = 8.26, *p* = 0.006, ɳp2 = 0.17]. Follow-up linear regressions showed that the magic subscale was a significant negative predictor of LBT errors in VR [β = − 0.39, 95%CI = − 0.69 to − 0.08, t(41) = − 2.55, *p* = 0.014] but not in the PR setting [β = 0.11, 95%CI = − 0.24 to 0.46, t(41) = 0.64, *p* = 0.53]. The other interactions were nonsignificant. Figure 4 shows this interaction.

## Discussion

The data showed that the laterality and direction of LBT bias in healthy participants differed in PR and VR conditions. In the PR setting, the bisection bias in most of the cases deviated toward the left hemispace depending on the degree of handedness. Stronger right-handedness is related to a stronger leftward LBT bias. The data from PR are congruent with other research results^32,37,39^. These results suggested that during the LBT-induced hemispace representation in PR environment, the visuospatial processing is related to elevated activation of the right hemisphere function. Due to the asymmetric visuospatial hemispheric activation, the attention allocation tends towards the contralateral hemispace and consequently, the left part of the visuospatial environment becomes overrepresented^55^. However, in the VR environment, the leftward LBT bias is decreased and drifted toward the right hemispace. The interpretation of this VR data currently has no other comparable data.

In addition, considering the role of the rate of right-handedness in both PR and VR conditions, the stronger handedness laterality is associated with a stronger LBT error bias towards the left hemispace. In contrast, a lower handedness score is associated with an LBT error bias drift towards the right hemispace. In other words, in participants with lower handedness scores in PR the left hemispace overrepresentation decreases and representation of the right hemispace improves. Ergo, in individuals with lower handedness scores, the left or right attention allocation and the laterality of the LBT bias tend to be equalised in PR. These results in PR condition are congruent with other studies indicating that ambiguous-handedness is originated from neurodevelopmental specificities and linked to lateralised dopaminergic hemispheric functions that are associated with a mixed functional dominance. This means that the semantic and visuospatial processing is shared between the left and right hemispheres^22,56,57^ that manifests in healthy individuals and numerous clinical cases^27,58,59^. Our results support the suggestion that handedness and lateralised visuospatial processing are primarily conditions for the execution of LBT^31^ that are related to weights of the visuospatial processing of stimuli in the right hemisphere in individuals with high right-handedness^60^.

On the other hand, considering the effect of the conditions, the presented results demonstrated that in VR compared to PR environment the LBT bias drifted toward the right hemispace similarly to individuals with lower handedness scores in PR condition. This means that the VR stimulation induces a balancing effect in the representation of the visuospatial hemispace. The rightward and the leftward attention allocation effect have been equalized. During VR stimulation the marked and articulated lateral visuospatial representation difference in the cognitive control of behavioral laterality (handedness effect) is reduced.

The main aim of this study was to reveal the role of schizotypy in visuospatial lateralisation of reality construction in a PR and a VR environment. To stimulate the mental construction of the visuospatial reality, VR device was used and LBT was employed to test the laterality of the hemispheric function. The PR data showed that SPQ-BR scales have no predictive values for the LBT bias; SPQ-BR-related significant LBT error bias laterality could not be detected. In addition, the results indicated that when participants performed LBT in VR, individuals with higher scores on the SPQ-BR cognitive schizotypy scale resulted in a more pronounced leftward bias than individuals with lower cognitive factor scores. In other words, VR-induced visuospatial reality construction in individuals with high cognitive (positive) schizotypy is associated with a left hemispace overrepresentation. This leftward pseudoneglect could originate from increased activity of the right-hemispheric reality construction. Similar schizotypy–related elevation of the right hemisphere activity in PR environment was not found. Therefore, schizotypy with a higher cognitive score (magical thinking, suspiciousness, and unusual perception) in PR conditions did not indicate specific right-hemispheric activity during reality construction. In contrast, in the VR condition, when the participant had not accounted for the real social context and the expected social dangers or negative feedback, being in VR the right-hemispheric activity worked with more functional articulation in the visuospatial context of realised actions. The pseudoneglect measured by LBT in schizotypy shows a conflicting pattern^59^ as schizotypy as a schizophrenia spectrum component, which is associated with structurally based reduced functional hemispheric lateralisation^8,13^. Previous studies used different asymmetry measurement and testing methods of schizotypy and either found a great variance or did not find significant bias in the leftward pseudoneglect^25,61,62^. Other studies on patients with schizophrenia found a prominent leftward bias^63^. However, there is still no consensus about the left or right-lateralised attention allocation bias in schizotypy, although most neurodevelopmental studies suggest a leftward bias in schizophrenia and positive schizotypy^64,65^.

The effect of ambiguous laterality manifests in handedness, speech and language processing, human face recognition, visuospatial processing, and attention allocation functions, causing a nonconventional view of the environment and reality construction^22,66^. This ambiguity balances hemispherically different cognitive functions where the left hemispheric activation engages with focused attention to semantically identifying (abstract categorisation, declarative functions) narrowed parts of the environment. In contrast, the right hemisphere functions spread in broader parts of the environmental context (action-oriented, procedural functions) and facilitate links between distant visuospatial associations^67^. The mentioned functions can be considered as a creative solution to this hemispheric functional leveling, but in the case of weakness of the self, and own thoughts perception account as a basic component in schizotypy and mainly in the magical thinking reality construction^15,68–71^.

Next, the study aimed to reveal the role of the SPQ-BR cognitive subscales in VR-related functional changes in the representation of scanned hemispace and the associated asymmetric hemisphere function. The results showed that magical thinking style had a pronounced role in the lateralisation effect in visuospatial construction of reality in VR, but a similar effect in PR condition was not found. Individuals with a high score on the magical thinking subscale showed an elevated leftward line bisection bias in VR conditions. That is, high magical thinking in VR is closely associated with the elevated right-hemispheric visuospatial functions. At the same time, high magical thinking is associated with relatively depressed left semantically-driven hemisphere functions. Overall, the SPQ-BR cognitive and magical thinking scores using VR application predict an overrepresentation in the left hemispace whilst the right hemispace is underrepresented. In PR conditions, similar effects were not detected.

Several theoretical frameworks may be relevant for the interpretation of the activation effect of VR in schizotypy. The biopsychosocial models of the brain laterality and the related functional asymmetries for schizotypy agree that atypical language lateralisation plays a role in ambiguous handedness but it is not limited only to the language areas and functions. The effect of the atypical lateralisation can be found in visuospatial and executive functions and manifests in the connection between the left and right hemispheres, visuospatial processing, visceral function, dopaminergic transmission, and genetically driven brain structural effect^32,64,72^. The atypical language representation exists with diffused semantic activation in the right hemisphere that induces magical thinking, delusions, suspiciousness, and creative achievements that frequently manifest in schizotypy^21,73,74^. FMRI examination with low versus high psychometrically measured schizotypy participants has shown that visuospatial originality and schizotypy show similar functional brain activity patterns (reduced deactivation of the right praecuneus) during creative problem solving^75^. However, the rate of schizotypy and visuospatial problems’ solution is associated with increased activation in the right prefrontal cortex^15^. This anterior and posterior conflicting duality and the related hyperdopaminergic activation in the striatum and the hypo-dopaminergic effect in the right prefrontal or right praecuneus^5,56^ may be an adequate interpretation frame for VR-induced correction effect on the right hemisphere. However, this is suggestions currently have not confirmed.

To summarise, VR technology has gradually increased clinical attention in the health care of schizophrenia spectrum disorders in the diagnosis of the suspiciousness symptom and other schizotypal traits^76,77^. In schizotypy, the VR seems to be an escape route from the real social context where the intended actions can be realised while the real social rules and feedback remain in the background of the problems solving activity. Our results have practical implications for designers who are considering using VR in neurocognitive rehabilitation for individuals with schizophrenia spectrum disorders. VR can be applied as non-invasive equipment to stimulate visuospatial processing in the right hemisphere. The presented data indicate that digitally generated and loaded visuospatial input can induce a correction in the imbalanced hemispheric functions, such as improving task-related articulation of the functional laterality in the hemispheres. However, the influence of the resulting visuospatial enhancement on other symptoms of schizotypy is currently unclear. In a healthy population, VR application improves prosocial skills and emotional empathy but the perspective-taking and a narrative component of empathy remain unchangeable^78^. In rehabilitation, VR used on patients with Parkinson’s disease could improve visuospatial functions (gait, orientation, and balance functions) similarly to dopamine elevation, but could also contribute to increased hallucinations^79^. It seems that VR plays a role in the activation of right-hemispheric functions but the effect of dopamine transmission on the cognitive phenotype of schizotypy is still unclear.

Based on the presented data it can be supposed that whilst a VR is induced, the degree of cognitive schizotypy has a marked role in the regulation of visuospatial representation and the laterality of the attention allocation in the digital environment. High cognitive schizotypy in part enhances the representation accuracy of left hemispace. While low cognitive schizotypy equalises the representational differences between left and right hemispace. Therefore, VR usage in schizotypy improves the articulation of cognitive regulation of the attention allocation that is controlled by right hemispheric function.

## Conclusion

The handedness and visuospatial reality construction were analysed in PR and VR environments. The outcome in the two simultaneous reality constructions was measured by an LBT that assessed the degree of the asymmetric hemispheric function. The atypical functional and structural asymmetries involving macrostructural, microstructural, and gene expression asymmetries^80^, and the so-called hemispheric imbalance^4^ are essential neurocognitive disorders in schizotypy and schizophrenia spectrum disorders and several other mental disorders but play a role in the neurodevelopmental plasticity to form unusual behavioral attitudes or cognitive talents as well^81,82^. The present study aimed to measure the schizotypal trait components that are usually associated with hemispheric imbalance. The restructuring of this imbalance may be an essential target for cognitive training for reality distortion and neurocognitive disorders. Our results highlighted that for individuals with schizotypy, the mental construction of a computer-generated VR could be an essential instrument to enhance the functional specification for the right hemisphere, and consequently improve visuospatial information processing and reality construction. In individuals with high cognitive schizotypy and related magical thinking, the VR-induced LBT error bias manifests on the left side of the hemispace. This hemispatial overrepresentation could be due to elevated activation in the right hemisphere. In contrast, in individuals with low cognitive schizotypy and low magical thinking, the LBT error bias drifts toward the right hemispace in VR. This could be due to rationally controlled left hemispace functions. From a rehabilitation point of view, it is unclear whether a highly immersed individual with cognitive schizotypy would be able to have training in semantical control over the invading visuospatial experiences and to obtain mental control over reality.

### Limitation

This study had a few limitations. Although the association between the mental construction of VR and the induced leftward hemispace overrepresentation is powerful, it needs to be replicated for final confirmation. Additionally, the question of whether the obtained experiences in VR could be transformed into a real environment and whether VR could contribute to the development of the participants’ mentalisation capabilities is currently unclear.

Another weakness, the distribution of self-reported hand dominance among males in an international population is right 87.4% and left 10.4%; among females 90.1% and left 8.4%^83^. The LBT error bias is differ when the response is performed in dominant or non-dominant hands. This study aimed to assess the LBT error in real and virtual environments only for right-handed individuals. Consequently, the presented results and interpretations refer to only right-handed individuals. The EHI^46^ has a long history. In clinical praxis, the EHI was usually used in numerous large sample investigations. Flowing this convention and the data comparability claims the usage of this simple assessment method in the present investigation. However, current examinations reveal numerous issues for classifying individuals as right or left-handed. New classifying methods are wanted. A current study^85^ recommends to the future researchers to improve the assessment of the degree of handedness: to control the actual version of the EHI; identify the difference between the EHI examination methods; to be aware of the limitation of the EHI usage. In this study, the original version of the EHI was used. However, it would be beneficial to use a detailed version of EHI that can descript the characteristics of inconsistent hand preference in a more detailed pattern. The degree of handedness bears on the cognitive performance, sleep quality, and other mental performance, talents, and psychopathological symptoms. Individuals with inconsistent handedness have benefits in visuospatial abilities (episodic memory), and right hemisphere-related procedural functions but the consistent handedness is associated with declarative processing and less time in REM sleep^84,85^. These cognitive and neurophysiological differences play an essential role in the regulation of the cognitive performances in attention allocation in LBT and the hemispheric functions of the brain. However, currently, the complexity in the integrative function of anterior and posterior and ventral and dorsal visual streams provides only a weak systematic prediction on the relationships between the schizotypy, the inconsistent hand preference, and lateralization-dependent cognitive performances.

## Data Availability

Link to a deposited file that contains all data of the study. https://data.mendeley.com/datasets/xch553k55w/1.
